# Angle-resolved X-ray emission spectroscopy facility realized by an innovative spectrometer rotation mechanism at SPring-8 BL07LSU

**DOI:** 10.1107/S1600577523010391

**Published:** 2024-02-01

**Authors:** Jun Miyawaki, Yuka Kosegawa, Yoshihisa Harada

**Affiliations:** a Institute for Solid State Physics (ISSP), The University of Tokyo, Kashiwa, Chiba 277-8581, Japan; b Synchrotron Radiation Research Organization, The University of Tokyo, Hongo, Bunkyo-ku, Tokyo 113-8656, Japan; Photon Science Innovation Center, Japan

**Keywords:** X-ray emission spectroscopy, resonant inelastic X-ray scattering, spectrometer

## Abstract

The X-ray emission spectrometer at SPring-8 BL07LSU has recently been upgraded with advanced modifications that enable the rotation of the spectrometer with respect to the scattering angle. This major upgrade allows the scattering angle to be flexibly changed within the range of 45–135°, which considerably simplifies the measurement of angle-resolved X-ray emission spectroscopy. To accomplish the rotation system, a sophisticated sample chamber and a highly precise spectrometer rotation mechanism have been developed. The sample chamber has a specially designed combination of three rotary stages that can smoothly move the connection flange along the wide scattering angle without breaking the vacuum. In addition, the spectrometer is rotated by sliding on a flat metal surface, ensuring exceptionally high accuracy in rotation and eliminating the need for any further adjustments during rotation. A control system that integrates the sample chamber and rotation mechanism to automate the measurement of angle-resolved X-ray emission spectroscopy has also been developed. This automation substantially streamlines the process of measuring angle-resolved spectra, making it far easier than ever before. Furthermore, the upgraded X-ray emission spectrometer can now also be utilized in diffraction experiments, providing even greater versatility to our research capabilities.

## Abstract 2

The X-ray emission spectrometer at SPring-8 BL07LSU has been upgraded for flexible spectrometer rotation, enabling angle-resolved measurements. This upgrade includes a sophisticated sample chamber, precise rotation mechanism and an integrated control system, streamlining measurements and expanding its usability.

## 1. Introduction

X-ray emission spectroscopy (XES) is a powerful technique to determine the energies of electronic states and elementary excitations in materials by irradiating X-rays onto a sample and analyzing the energy of the emitted X-rays from the sample. By utilizing the momentum of the incident X-ray, angle-resolved XES measurements enable us to investigate the dispersion relation between energy and momentum. Recent advances in energy resolution have enabled ultrahigh energy resolution below 50 meV at the Cu *L*-edge (Brookes *et al.*, 2018 ▸; Zhou *et al.*, 2022 ▸), allowing for the observation of magnons and phonons. XES has become an important technique for observing low-energy excitations in a complementary manner to inelastic neutron scattering (Kotani & Shin, 2001 ▸; Ament *et al.*, 2011 ▸). The pioneering work on the SAXES spectrometer at the ADRESS beamline of the Swiss Light Source demonstrated the significance of spectrometer rotation for measuring the angular dependence of momentum and observing magnon dispersion in XES (Strocov *et al.*, 2010 ▸; Braicovich *et al.*, 2009 ▸). Since then, most XES spectrometers constructed after SAXES have been equipped with a spectrometer rotation feature (Brookes *et al.*, 2018 ▸; Zhou *et al.*, 2022 ▸; Dvorak *et al.*, 2016 ▸; Singh *et al.*, 2021 ▸; Schulz *et al.*, 2020 ▸; Chuang *et al.*, 2022 ▸).

The XES spectrometer installed at the HORNET end­station of SPring-8 BL07LSU has been a valuable tool for understanding the electronic and chemical properties of materials (Yamamoto *et al.*, 2014 ▸; Harada *et al.*, 2012 ▸). However, its fixed scattering angle of 2θ = 90° limited its capabilities to provide a comprehensive analysis of materials. To overcome this limitation, we implemented a spectrometer rotation function without breaking the vacuum, enabling us to take angle-resolved XES measurements at any scattering angle. Our goal was to develop a convenient and efficient system by taking advantage of the relatively small size of the spectrometer. By rotating the spectrometer smoothly and accurately, we aimed to eliminate the need for optical adjustments after the rotation and make angle-resolved XES measurements more facile. The rotation range of 2θ was set to 45–135°, taking into consideration the configuration of the existing equipment.

In this paper, we provide an overview of the HORNET optical system, the outline of the spectrometer rotation system, performance evaluation and an example of angle-resolved XES measurement. Our upgraded XES spectrometer offers a unique opportunity to gain insightful information on the electronic and chemical properties of materials, and we believe that it will be an important tool for advancing our understanding of materials science.

## 2. Optical system of the HORNET spectrometer

**Figure 1:**
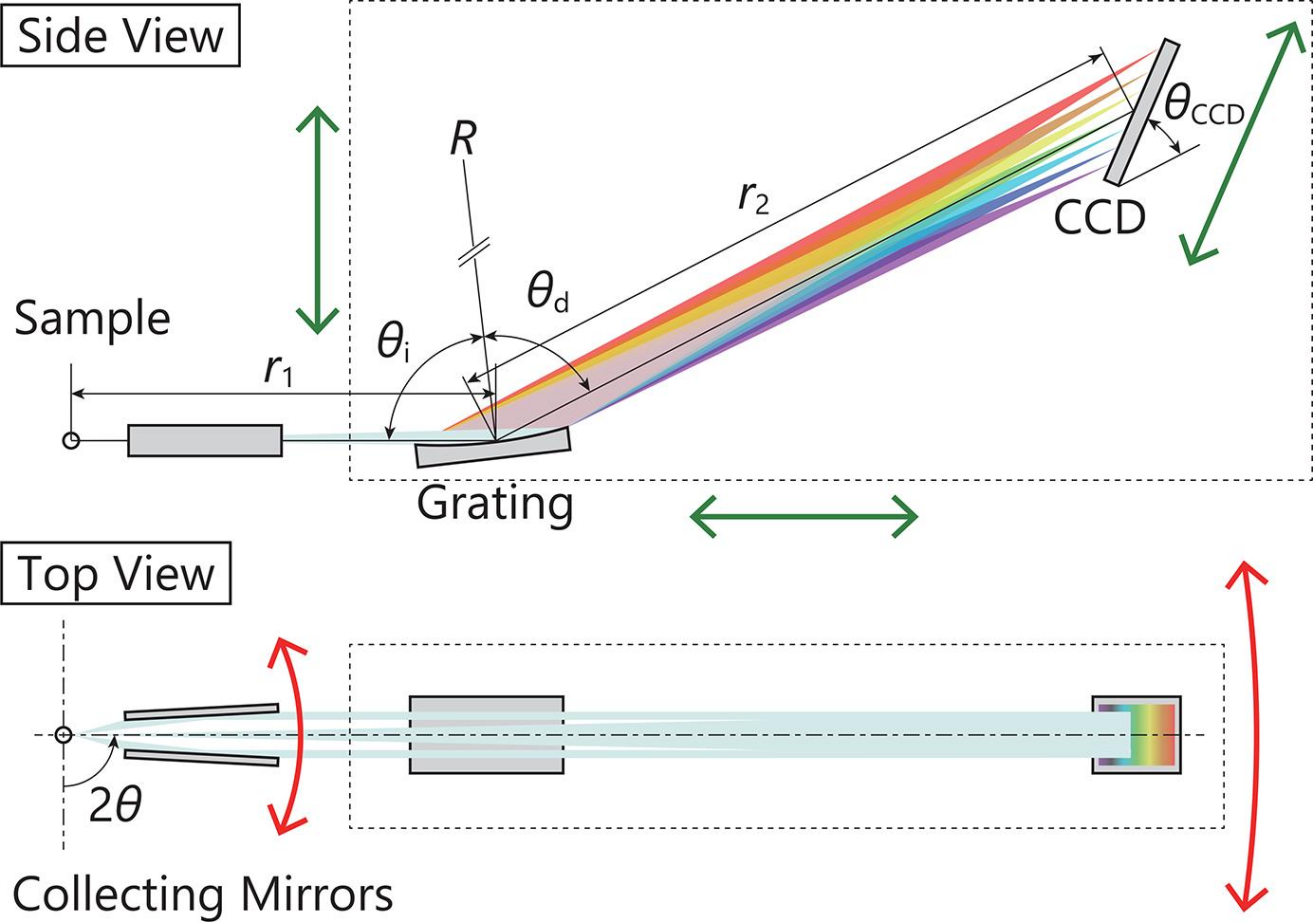
Optical layout of the XES spectrometer of HORNET. The spectrometer disperses the X-rays vertically using a diffraction grating and detects the energy dispersion in a single shot with a CCD to obtain XES spectra. Two cylindrical collecting mirrors are installed near the sample to increase the acceptance angle in the horizontal plane. The green and red arrows indicate the motion axes for the optical optimization and the direction of spectrometer rotation, respectively. The spectrometer rotation angle 2θ is set to be 0° for forward scattering and 180° for back scattering. The dashed rectangle indicates that the grating and CCD stages are mounted on the same frame.

The X-ray emission spectrometer used in HORNET is composed of a varied-line-spacing (VLS) cylindrical grating and a CCD detector. When X-rays are emitted from a sample, the grating energetically disperses them, and the energy-dispersed X-rays are then detected by the CCD to obtain XES (Fig. 1 ▸). This simple optical system, which includes only the grating and detector, has been widely adopted in many XES spectrometers because of its high efficiency and the minimal number of optical elements that need to be adjusted (Ghiringhelli *et al.*, 2006 ▸; Strocov *et al.*, 2011 ▸). To further improve the throughput of HORNET, two cylindrical mirrors are installed near the sample, oriented perpendicular to the grating in the XES spectrometer, to increase the horizontal acceptance angle without affecting the energy resolution of the spectrometer (Tokushima *et al.*, 2011 ▸). The total distance from the sample to the CCD is approximately 2.5 m, and the grating and CCD stages are mounted on the same rigid frame.

The optical system of the XES spectrometer is determined by several parameters: the distance *r*_1_ from the sample to the grating, the distance *r*_2_ from the grating to the CCD, the incident angle θ_i_ and diffraction angle θ_d_ of the grating, and the incident angle θ_CCD_ to the CCD. In HORNET, θ_i_ is adjusted by vertically moving the spectrometer, rather than rotating the pitch angle of the grating, and the CCD can only be moved along the direction of the focal plane of the grating obtained from simulation. This simplifies the optical adjustment process and improves stability. Therefore, the axes used to optimize the HORNET spectrometer are *r*_1_, θ_i_ and θ_d_. However, since θ_d_ is determined by the energy of the emitted X-ray to the spectrometer, the optical performance of the system is determined only by *r*_1_ and θ_i_. Since the grating and CCD stages are mounted on the same frame, the position relationship between them is not affected by changes in *r*_1_ and θ_i_ and by the rotation of the spectrometer. Once the spectrometer is optimized, the positions of *r*_2_, θ_d_ and θ_CCD_ are maintained with high mechanical stability, which eliminates the need for recalibration after the spectrometer rotation.

There are three paths through which the emitted X-rays from the sample reach the CCD: the direct path, where the X-rays enter the grating directly without being reflected by the collecting mirrors, and two paths where the X-rays reflect off two collecting mirrors before entering the grating. These latter paths, which we refer to as the Trident paths, require that the collecting mirrors be orthogonal to the grating in the spectrometer to prevent affecting the energy resolution of the spectrometer. To ensure this, the two collecting mirrors are mounted on the same stage, guaranteeing that they are parallel to each other. The collecting mirrors are then rotated around the direct optical path to optimize the mount angle for multiple gratings.

In order to determine the necessary accuracy for the collecting mirrors and the X-ray emission spectrometer (consisting of the grating and CCD) when the spectrometer was rotated, we employed ray-tracing simulation with *SHADOW* software (Sanchez del Rio *et al.*, 2011 ▸). The ray-tracings for the direct and Trident paths were performed separately, and the intensities were combined to account for the reflectivity of the collecting mirrors and reproduce the actual measurements. The criteria for required accuracy were set allowing for a small energy shift as long as the energy resolution was not degraded. The energy shift was not problematic, as it could be converted to energy loss based on the position of the elastic scattering peak. The ray-tracing simulation was performed at 640 eV, a high-energy reference, as shown by Harada *et al.* (2012 ▸). The parameters of the spectrometer are summarized in Table 1 ▸.

After conducting ray-tracing simulations, we identified that the height position or pitching angle of the spectrometer has a significant impact on energy resolution and shift. However, we found that mechanical shifts in other axes have a large tolerance and can practically be considered negligible, as they would not significantly impact the energy resolution or shift. Specifically, change in the height of the spectrometer by 20 µm or change in the pitch angle by 0.0023° cause the resolution to degrade by approximately 10%. Although these changes also cause the position shift of the whole image on the CCD by around 120 µm, this energy position shift can be converted to loss energy based on the position of the elastic peak, making the energy shift a non-issue.

**Figure 2:**
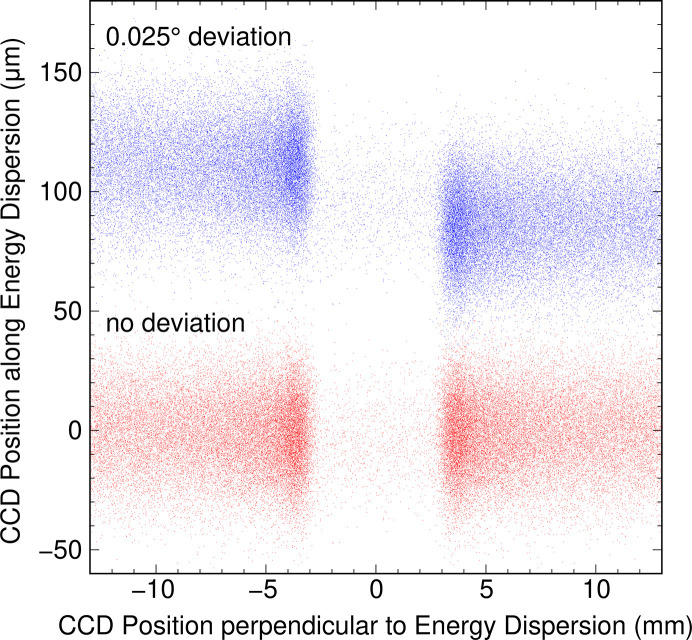
Results of ray-tracing simulation for the XES spectrometer at 640 eV. The red and blue dots indicate the rays on the CCD when the collecting mirrors is orthogonal to the grating of the spectrometer and when their roll angles are rotated by 0.025°. Two results are offset by 100 µm along the ordinate to improve visibility.

In the case of the collecting mirrors, it was found that the roll angle (rotation around the direct optical path) has a significant impact on the energy resolution, requiring a mechanical shift of less than 0.01°. Fig. 2 ▸ shows a comparison of ray-tracing results at the CCD between for the optimal position of the collecting mirrors and with a shift of 0.025° in roll angle. It is important to note that this figure only displays the ray-tracing position on the detector and does not include information on intensity changes due to the reflectivity of the collecting mirrors, which is considered when evaluating the resolution by integrating intensity. The image detected by the CCD becomes blurred due to the spatial resolution of the detector. The density of the rays around 0 along the abscissa of Fig. 2 ▸ is low, but it becomes high on both sides. This increase in ray density is due to the detection of X-rays from the two Trident paths, while the low density near 0 is due to the detection of only X-rays from the direct path. It is worth noting that X-rays from the direct path are present across the entire region, not just at the center. However, these X-rays overlap with those from the Trident paths, making them difficult to discern. When the roll angle of the collecting mirrors is shifted by 0.025°, the image from the Trident paths on both sides rotates and shifts away from the image from the direct path. As the resolution of the spectrometer is evaluated by integrating intensities along the abscissa, the resolution deteriorates due to the deviation of the roll angle from the optimal position.

In the development of the spectrometer rotation system, the 2θ rotations of both the spectrometer and collecting mirrors were designed with attention to meeting their respective requirements.

## 3. Spectrometer rotation system

This section describes the spectrometer rotation system that comprises a measurement sample chamber capable of moving the vacuum flange that connects to the spectrometer to any desired scattering angle and continuously tracking the rotation of the spectrometer, a rotation mechanism that enables high-precision 2θ rotation of the spectrometer, and a control and interlock system that comprehensively manages the rotation of the spectrometer, beamline and sample manipulator, allowing seamless data acquisition under vacuum condition. In the following subsection, details about the sample chamber and the rotation mechanism will be provided, while an overview of the control system can be found in the supporting information.

### 3.1. Sample chamber

The sample chamber must fulfill several critical requirements to ensure successful operation of the spectrometer rotation. Firstly, the chamber must stably hold the sample in place during spectrometer rotation while also allowing for precise position adjustment in a vacuum environment. Secondly, the vacuum flange connecting the spectrometer must be capable of a wide angle range of motion without breaking the vacuum. This is vital to enable emitted X-rays from the sample to reach the spectrometer at any desired scattering angle within the 2θ range of 45–135°. Additionally, collecting mirrors installed in the sample chamber must rotate with the spectrometer as it moves between the 2θ angles of 45 and 135°. The sample chamber must meet all of these requirements within a diameter of approximately 600 mm, due to space constraints imposed by surrounding components. Given these constraints, installing a mechanism for rotating the collecting mirrors in the chamber was deemed impractical. Instead, the collecting mirrors were mounted on the connecting flange of the sample chamber.

**Figure 3:**
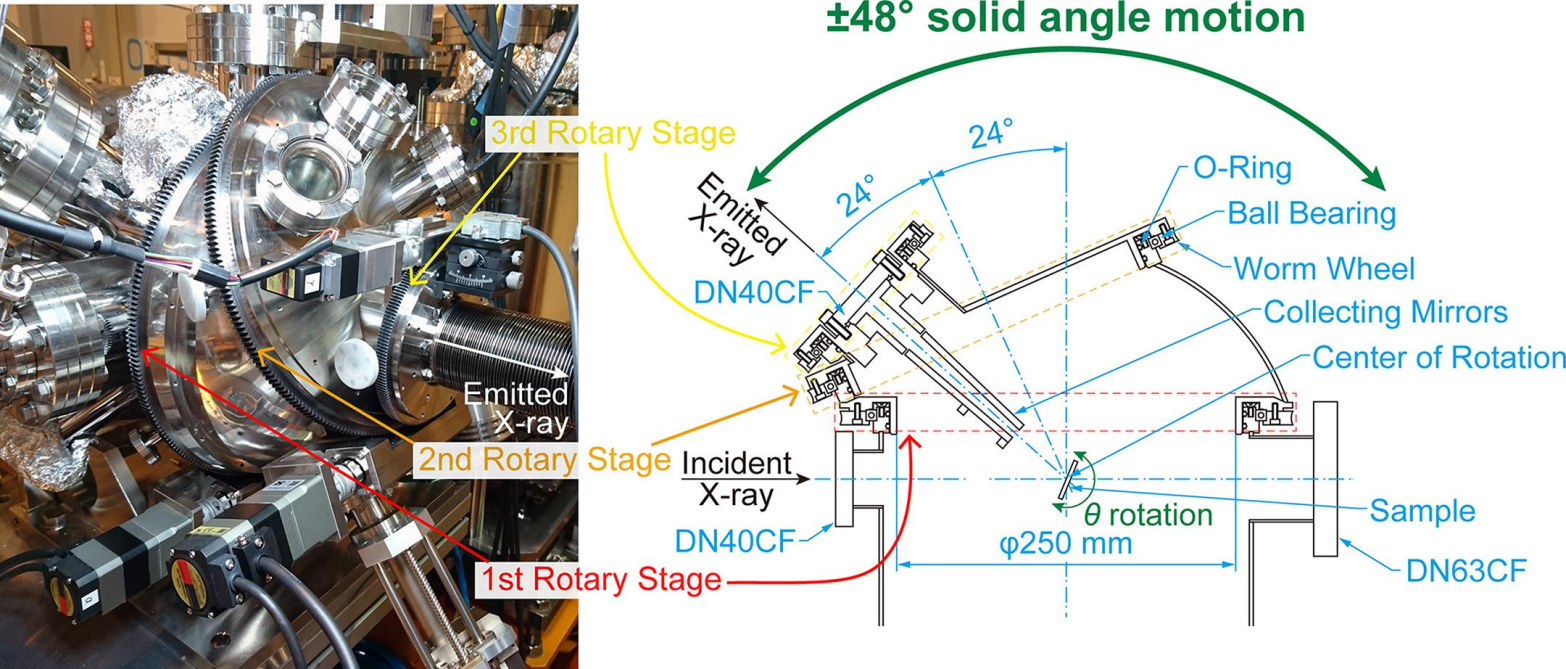
Photograph and schematic representation of the sample chamber equipped with the rotation mechanism of the vacuum flange, which is connected to the spectrometer and follows the 2θ rotation. 2θ is 90° and 138° in the photograph and schematic representation, respectively. The sample can be rotated as θ rotation, and its center of rotation can be aligned with the center of the 2θ rotation.

Fig. 3 ▸ shows the intricate mechanism of the sample chamber developed for this system. The mechanism consists of three rotary stages. The first rotary stage is mounted on the chamber, and the position is always fixed. On the other hand, the second and third rotary stages are mounted on the first and second rotary stages with a tilted angle, and thus the positions change in response to the rotation of the first and second rotary stages, allowing the connecting flange to rotate widely tracking the 2θ rotation of the spectrometer. Both the rotation axes of the first and second rotary stages intersect at the center of the spectrometer rotation, and the angles between the rotation axes of the first and second rotary stages and between those of the second and third rotary stages are arranged to be the same. By combining the rotation angles of the first and second rotary stages, the position of the connecting flange can be changed three-dimensionally over a wide angle range. The third rotary stage is equipped to compensate for the twisting of the connecting part. The collecting mirrors are designed to be mounted on the third rotary stage. If we only consider the vacuum connection between the sample chamber and the spectrometer, a slight deviation in the position of the flanges is not a significant issue because it can be absorbed by the bellows between the flanges. Therefore, the rotation mechanism of the sample chamber can also be used to fine-tune the 2θ position and roll angle of the collecting mirrors.

Various rotation mechanisms can be used in sample chambers for spectrometer rotation, as demonstrated by other facilities such as ERIXS at ESRF, Veritas at MAX IV and AGM-AGS at TPS (Brookes *et al.*, 2018 ▸; Englund *et al.*, 2015 ▸; Lieutenant *et al.*, 2016 ▸; Singh *et al.*, 2021 ▸; Dvorak *et al.*, 2016 ▸). However, our sample chamber’s rotation mechanism offers the unique advantage of compactness. Furthermore, while a one-dimensional movement on the scattering plane is sufficient for the sample chamber to follow the spectrometer rotation, our sample chamber has the capability to move the flange in three dimensions, allowing it to serve as a versatile adjustment mechanism for the collecting mirrors, as mentioned above.

The following explains how to calculate the angles of the three rotary stages required to move the connection flange to the desired position.

**Figure 4:**
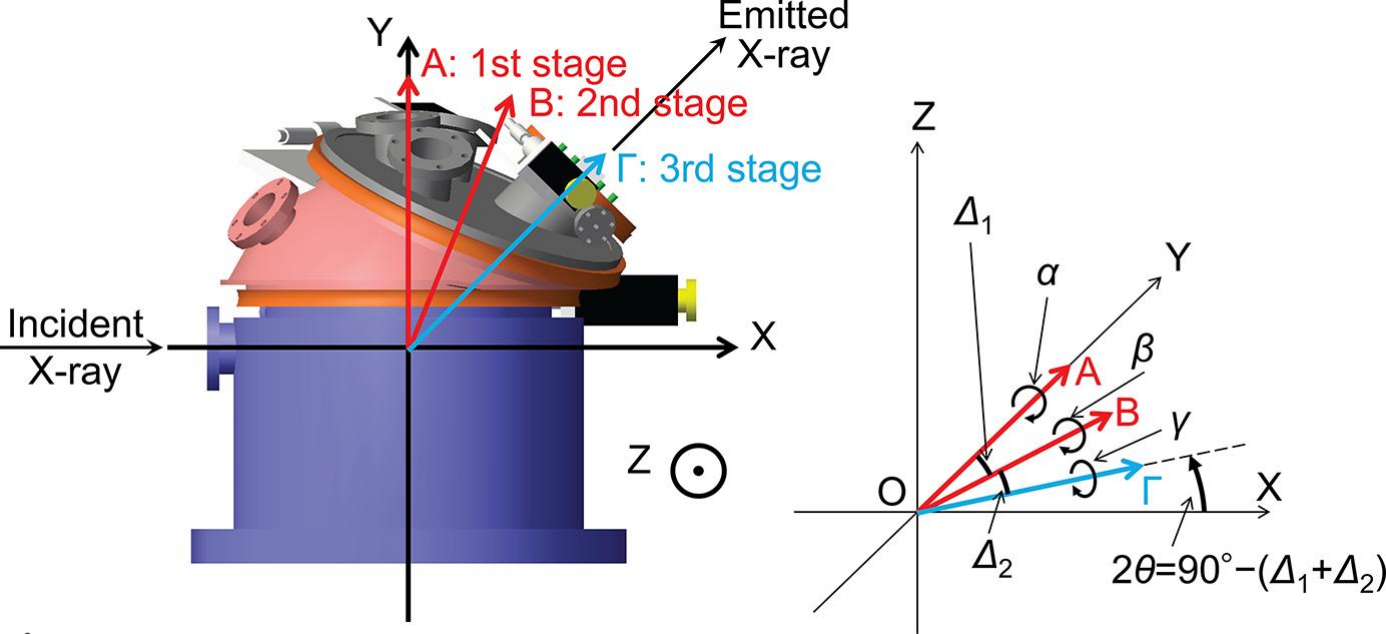
(Left) Definition of the rotation axes *A*, *B* and Γ for the first, second and third rotary stages, as well as the definition of the *XYZ* coordinates. The *X* axis aligns with the incident X-rays, the *XY*-plane corresponds to the horizontal scattering plane, and consequently the *Y* axis is along the direction of 2θ = 90°. The Γ axis (the direction of the connecting flange) should align with the spectrometer to pass the emitted X-rays. (Right) Definition of the angles Δ_1_ and Δ_2_ between the *A* and *B* axes and *B* and Γ axes, respectively. Additionally, the direction and 0° position of rotation α, β and γ around the three axes *A*, *B* and Γ are defined. At α = β = γ = 0°, 2θ becomes 90° − (Δ_1_ + Δ_2_).

The definitions of the *XYZ* coordinates, the position relationships of each rotation axis, and the rotation directions are shown in Fig. 4 ▸. The origin of the coordinates is at the center of rotation. *A*, *B* and Γ indicate the rotation axes of the first, second and third rotary stages, respectively. Δ_1_ and Δ_2_ represent the angles between the rotation axes of the first and second rotary stages and between the second and third rotary stages, respectively. α, β and γ denote the rotation angles of the first, second and third rotary stages. The *A* axis is always fixed and along the *Y* axis, while *B* and Γ axes change their positions in response to the angles α and β. The position of the flange for the incident X-ray is fixed, and the emitted X-ray passes through the flange along the Γ axis. The 0° position and direction of rotation for each angle are as shown in Fig. 4 ▸. The *A*, *B* and Γ axes are on the *XY* plane.

**Figure 5:**
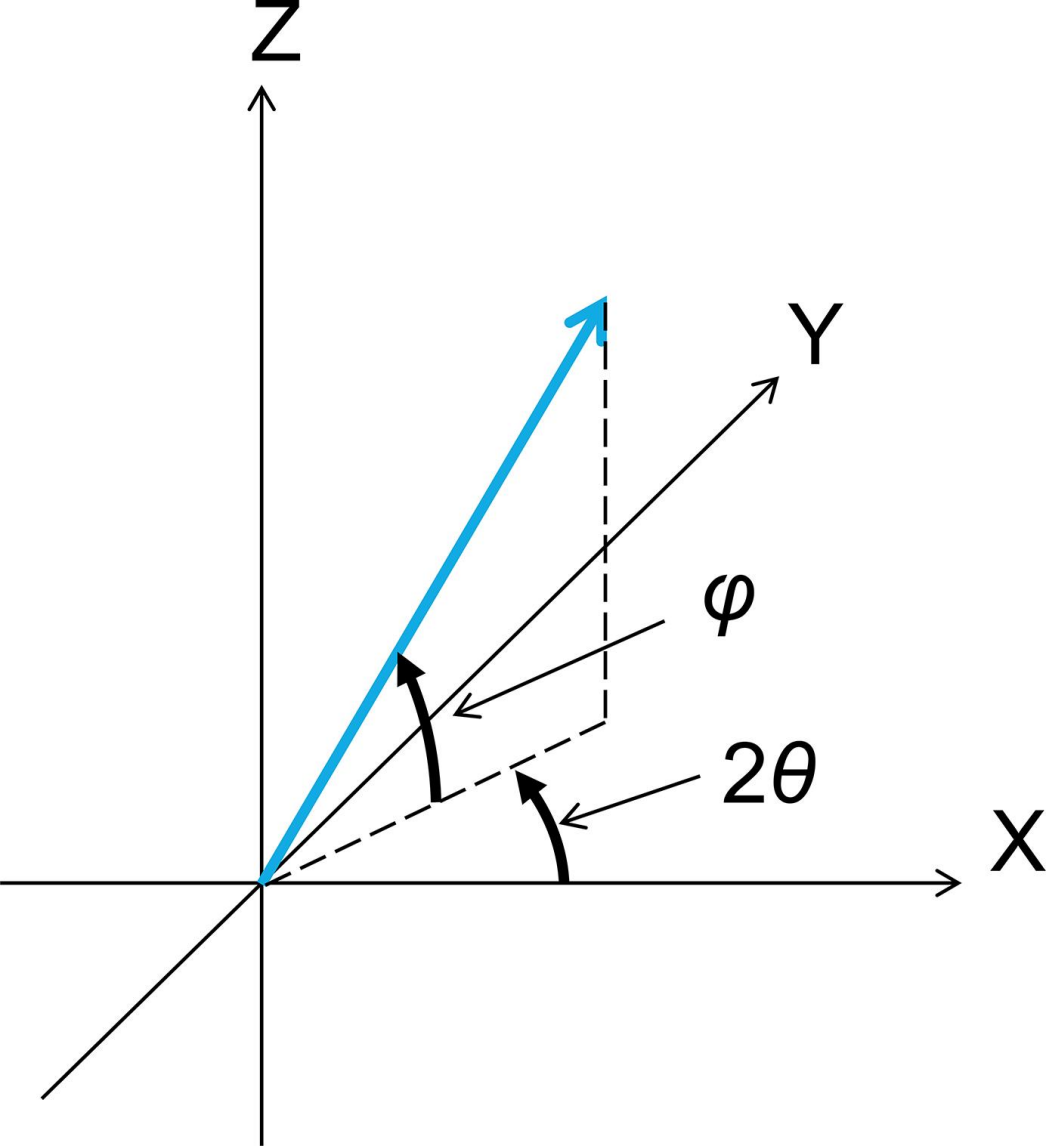
Definition of 2θ and φ to represent an arbitrary position of the Γ axis on a sphere from the viewpoint of the experimental configuration. The definition of the *XYZ* coordinates is the same as for Fig. 4 ▸. The blue arrow indicates the direction of the Γ axis, which can be changed by rotating around the *A* and *B* axes, corresponding to the blue arrows in Fig. 4 ▸. 2θ and φ are defined as the azimuth angle from the *X*-axis on the *XY* plane and the elevation angle from the *XY* plane, respectively.

To move the Γ axis to an arbitrary position on a sphere, as shown in Fig. 5 ▸, the required α and β can be determined using the following formulas,

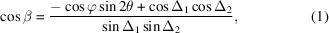




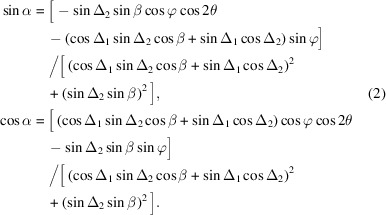

In order to cover the whole range of 90° − (Δ_1_ + Δ_2_) < 2θ < 90° + (Δ_1_ + Δ_2_), Δ_1_ = Δ_2_ is required. To prevent any twist in the angle between the connection flange of the sample chamber and the flange of the spectrometer, the third rotation axis, Γ, must be rotated to satisfy the following formula,

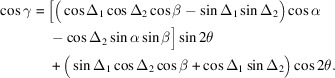

**Figure 6:**
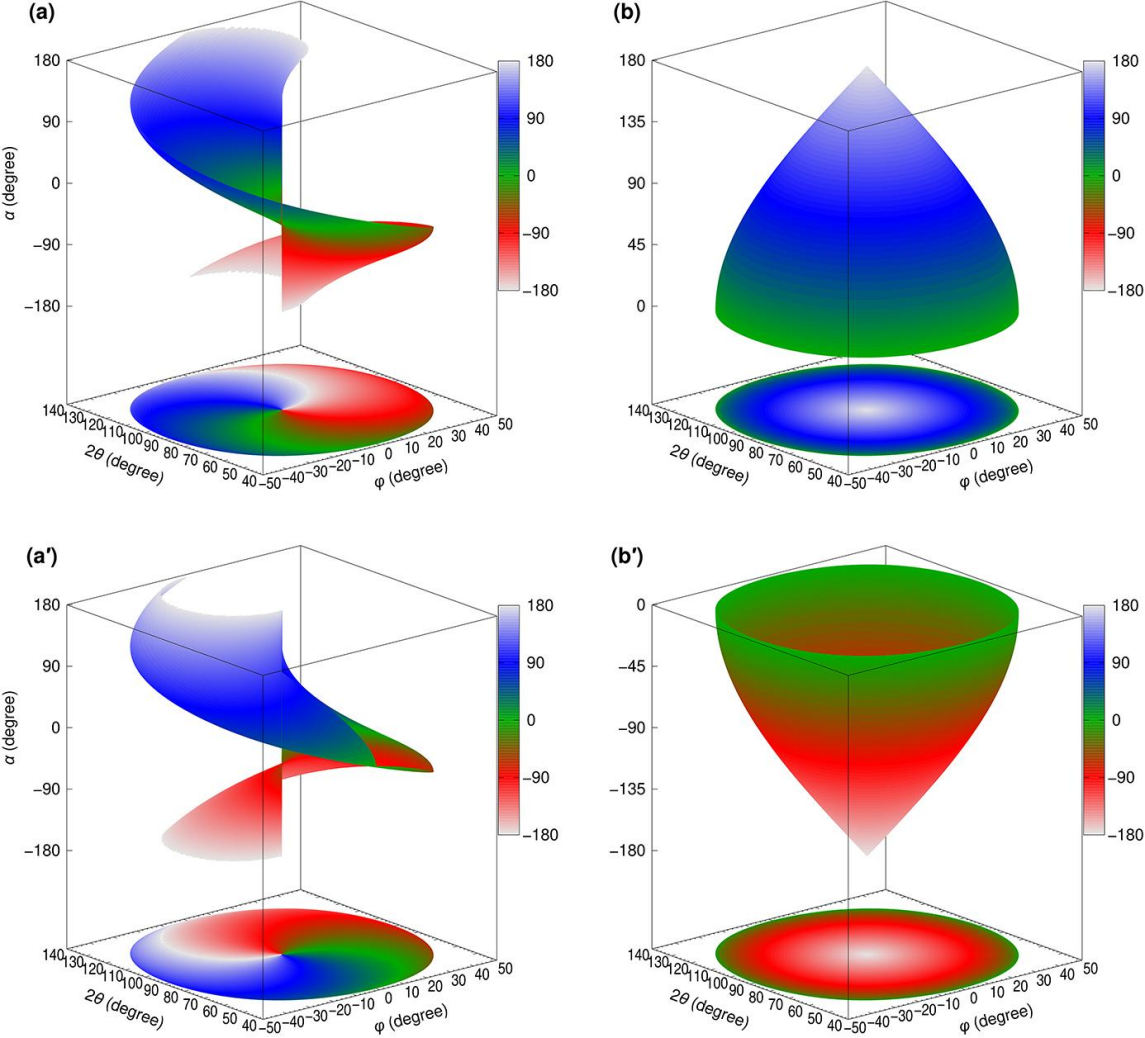
Calculated α and β required for full range motion of 2θ and φ when Δ_1_ = Δ_2_ = 24°.

**Figure 7:**
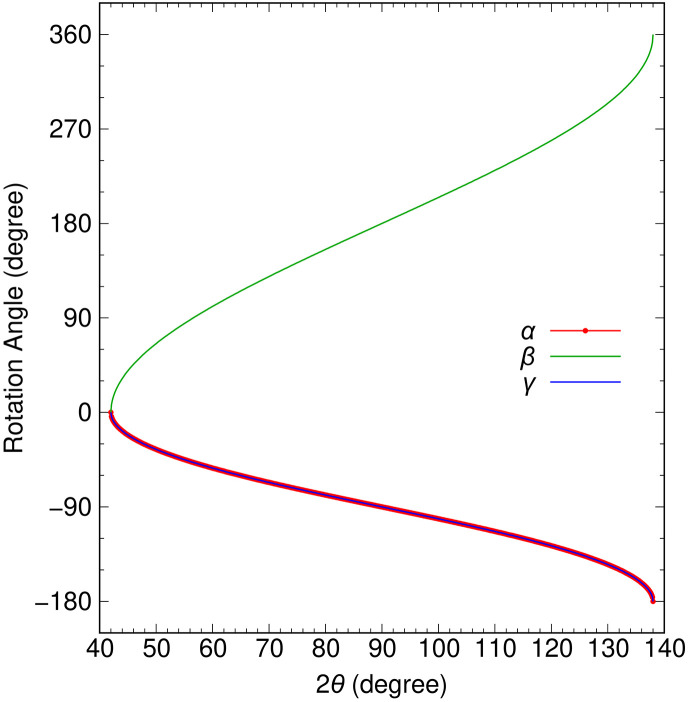
Calculated α, β and γ required to move over the range of 2θ = 42–138° at φ = 0 when Δ_1_ = Δ_2_ = 24°.

Fig. 6 ▸ shows the calculation results for the required values of α and β when using Δ_1_ = Δ_2_ = 24°, which is the configuration used in our sample chamber. These results demonstrate that the Γ axis can move freely over the entire three-dimensional solid angle of ±48°. For any given combination of 2θ and φ, two patterns of α and β satisfy equations (1) and (2). These patterns are shown separately at Figs. 6 ▸(*a*) and 6 ▸(*a*′) and Figs. 6 ▸(*b*) and 6 ▸(*b*′) for clarity, but both patterns are equally viable and can be used interchangeably. The two patterns are connected at the outer circumference and center point (2θ = 90° and φ = 0°), where switching between the patterns is possible. The outer circumference corresponds to α rotating by 360° with β at 0° (or 360°), and α and β are uniquely determined. The center point corresponds to β at 180°, where the *A* and Γ axes are aligned, and it is a singularity point where 2θ and φ are always 90°and 0° for any value of α. This is known as ‘gimbal lock’, and special attention is required when operating the system near this point. Using only one pattern results in a discontinuous angular motion, but, by switching between both patterns at the center point, continuous angular motion can be achieved. Fig. 7 ▸ shows the values of α, β and γ required for the spectrometer rotation, which corresponds to the motion of changing only 2θ at φ = 0. As mentioned earlier, switching between the two patterns at 2θ = 90° results in continuous angular motion.

The purpose of γ is solely to prevent twisting between the connection flanges and is not related to the adjustment of 2θ and φ. As 2θ and φ can be adjusted independently of γ, it is possible to use γ for fine-tuning the roll angle of the collecting mirrors to align the Trident paths shown in Fig. 2 ▸ by slightly changing the γ angle from the calculated value in Fig. 7 ▸. The deviation of the Trident paths is caused by the misalignments of the roll angles of the two gratings in the spectrometer with respect to the scattering plane, and the difference in these misalignments are ∼0.5°. Therefore, the slight change of the γ angle falls within the mechanical tolerance of the bellows at the connection. At HORNET, the spectrometer has a 2θ range of 45–135°, which could be covered even with Δ_1_ = Δ_2_ = 22.5°. However, as shown in Fig. 7 ▸, the change in β near the outer circumference becomes very steep, making it difficult to move. To avoid steep changes in β and provide sufficient margin for the rotation range, we have set Δ_1_ = Δ_2_ = 24° to cover 2θ = 90 ± 48°.

### 3.2. Precise rotation of the spectrometer

**Figure 8:**
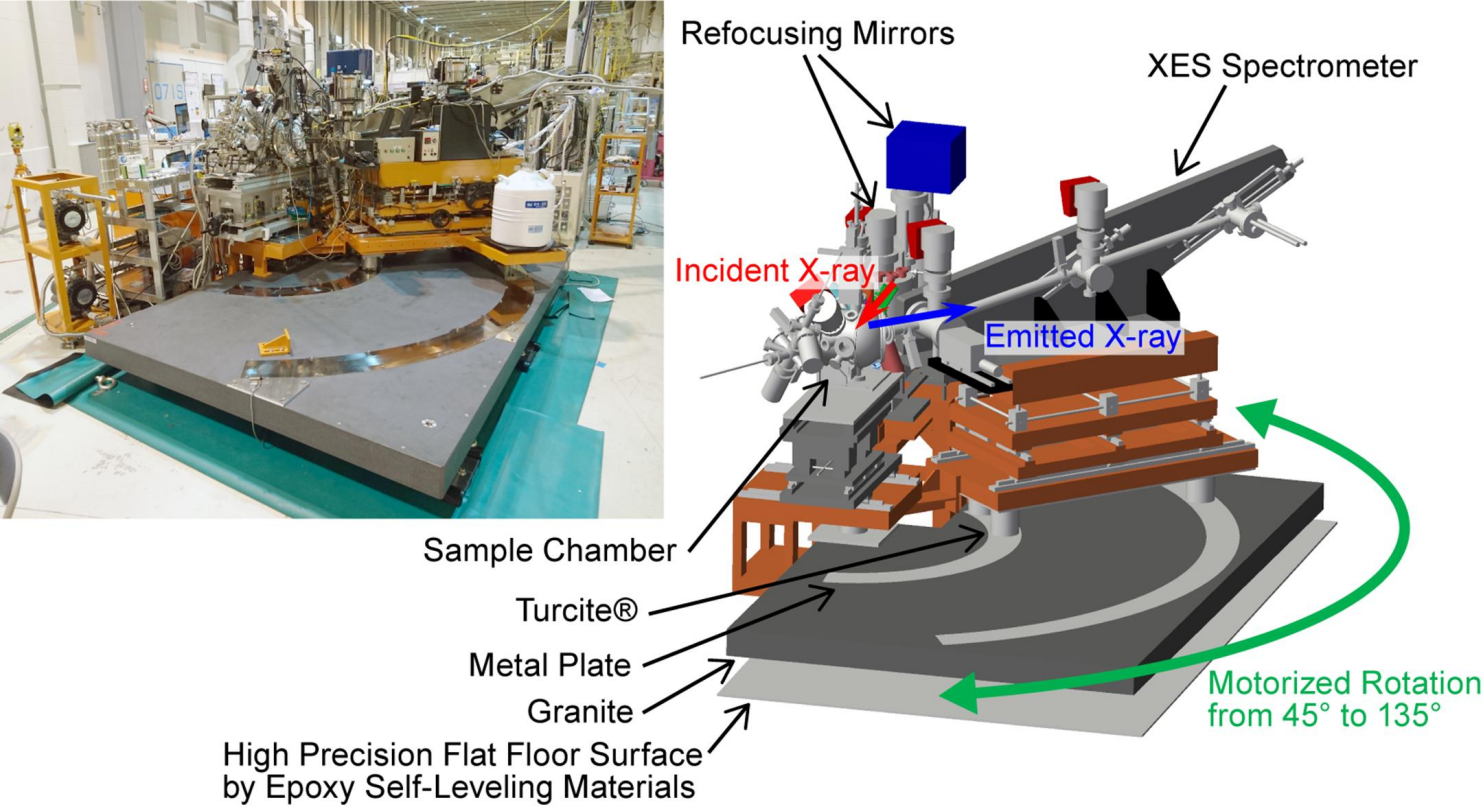
Overview of the spectrometer rotation system. Photograph (left) and schematic representation (right) of the entire system at 2θ = 135°.

Fig. 8 ▸ displays a photograph and schematic representation of the rotation mechanism used in the HORNET spectrometer. Kirkpatrick–Baez mirrors are used to refocus the X-rays onto the sample, and, since the angle of incidence for the vertical focusing mirror is 89°, X-rays are incident on the sample at an angle of 2° from the horizontal plane. To compensate for this angle and attain accurate measurements, the rotation plane must be tilted by 2°. This was achieved by installing a granite base on which the spectrometer rotates, tilted by 2°. To ensure high accuracy and minimize vibrations, the granite base was placed on a self-leveling ep­oxy resin floor with a high level of flatness. As a result, the granite base serves as the precise rotation platform for the spectrometer. Although air pads are often used to move heavy objects smoothly by slightly floating them on a cushion of air, the spectrometer rotation system at HORNET rotates the spectrometer by sliding on the metal plates while maintaining contact with them. This innovative approach maintains a constant height position of the spectrometer during rotation, allowing for measurements during rotation if the mechanical accuracy and intensity of the spectrum are sufficient.

We measured the installation and rotation accuracy using a laser tracker. To evaluate the flatness of the metal plates, we measured the height deviation from the rotation plane (a plane tilted 2° from the horizontal plane) at 5 and 13 points on the inner and outer metal plates, respectively. The variations were 7.4 µm and 9.0 µm in standard deviation (SD) and 18 µm and 39 µm in peak-to-valley (PV) for the inner and outer plates, respectively. If the spectrometer rotates with this accuracy, which does not exceed the resolution degradation limit of 20 µm, optical readjustments will not be required during rotation. Next, we rotated the spectrometer in 5° increments in the entire range of 2θ = 45–135° and evaluated the shift of the rotation center, the height deviation near the diffraction grating, and pitch angle deviation. The shift of the rotation center along the optical axis was very small, with SD and PV values of 26 µm and 83 µm, respectively. These values are well below the 400 µm limit where energy shift and degradation of energy resolution may occur, so the rotation centers are accurate enough. The height variation near the diffraction grating was 5.4 µm in SD and 19.4 µm in PV, while the pitch variation was 0.00052° in SD and 0.0019° in PV, both meeting the accuracy requirements of 20 µm for height and 0.0023° for pitch angle. Thus, we have confirmed the ultrahigh precision capability of our spectrometer rotation system using the laser tracker, indicating that angle-resolved XES measurements can be performed conveniently without requiring any spectrometer adjustments.

## 4. Performance evaluation of spectrometer rotation system

To evaluate the performance of the spectrometer’s rotation mechanism, we conducted a performance evaluation by acquiring spectra after rotating the spectrometer. We measured elastic scattering from a copper plate at the oxygen *K*-edge and analyzed the full width at half-maximum (FWHM) and energy position of the resulting spectra. The spectrometer’s rotation angle was varied incrementally by 5° steps from 45° to 135°. After each rotation, we performed only the necessary adjustments to the 2θ and roll angles of the collecting mirrors, without making any adjustments to the spectrometer itself.

**Figure 9:**
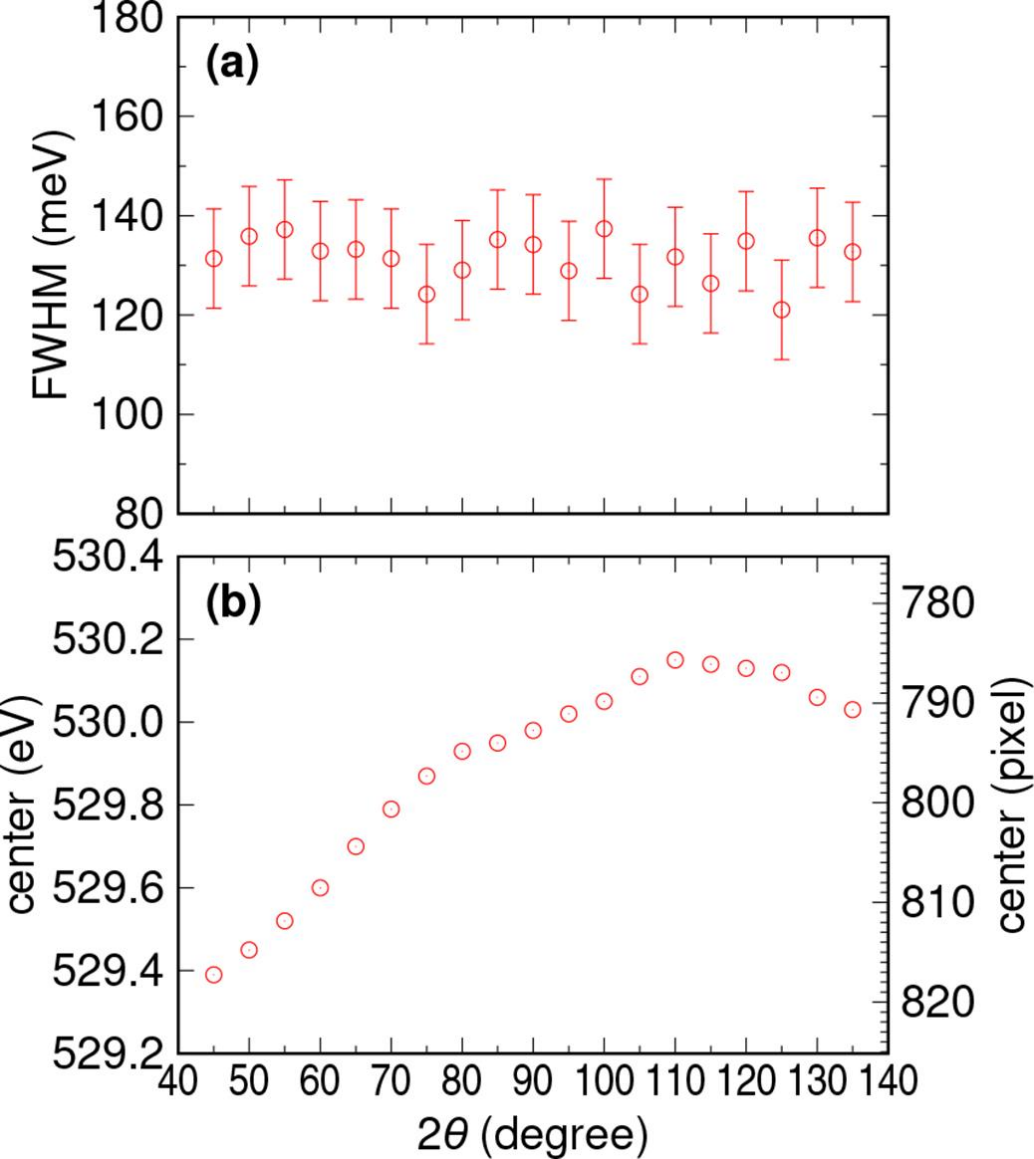
Results of the performance evaluation of the spectrometer rotation. Elastic scattering from a copper plate was measured at the O *K*-edge at each 2θ angle, and (*a*) the FWHM and (*b*) the peak position were evaluated. In the entire 2θ range, the γ angle was set −0.15° away from the calculated value to align the Trident paths.

Fig. 9 ▸ presents the results of the performance evaluation. As expected from the mechanical precision, the resolution was excellent across the entire 2θ range without any degradation. However, there was a shift in the emission energy of approximately ±10 pixels = ±135 µm, which falls within the estimation from the measured mechanical precision. Given the size of the CCD (∼25 mm × ∼25 mm), detection is not a concern, and the energy shift can be calculated based on the position of the elastic scattering and converted to loss energy, making it a non-issue. The 2θ and roll angles of the collecting mirrors did not reach the optimal position as originally designed. Nonetheless, since the reproducibility of the deviation from the optimal position is high, corrections can be made. Regarding the vacuum pressure of the sample chamber, the base pressure is typically around 1 × 10^−6^ Pa when the chamber is used without baking and is not degraded due to the rotation. Thus, there is no need for any adjustment of the collecting mirrors or the spectrometer after the rotation. The primary objective of convenient and straightforward angle-resolved XES measurement is attainable.

The evaluation highlighted the importance of accurately determining the absolute angle of the three rotation axes for achieving precise positioning of the collecting mirrors. To achieve the 2θ motion with the three rotary stages, it is necessary to control three rotations with the curved relationship as shown in Fig. 7 ▸. While the right-hand image in Fig. 4 ▸ defines the 0° positions for α and β, if these 0° settings are incorrect it becomes impossible to move the connecting flange to the correct position. Furthermore, since the pulse control of the three rotations are not linear but rather curved, the offset from the correct position does not remain constant with respect to 2θ; instead, it varies depending on 2θ. This makes the control more complex, as the fine-tuning by the slight change in the γ angle varies with 2θ, and if the deviation cannot be corrected it adversely affects measurement accuracy and resolution. To address these difficulties, we can determine the angle of α and β by the following method: first, determine the angle of β by the fact that the *A* and Γ axes coincide at β = 180°; next, we can determine the angle of α by the fact that the *B* axis is either parallel or perpendicular to the scattering plane at α = 0 or ±90°, respectively.

**Figure 10:**
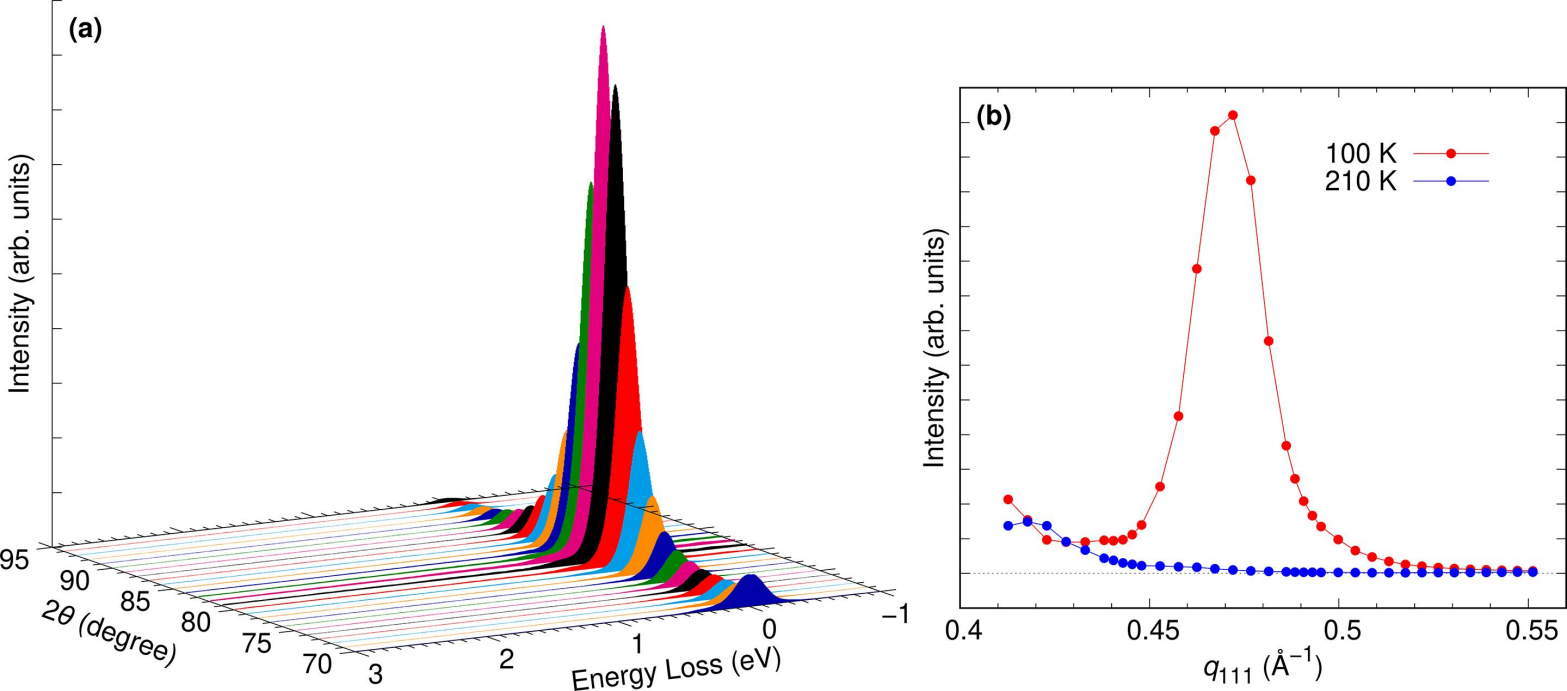
Example of θ–2θ measurement using the spectrometer rotation system. The Fe *L*-edge REXS of La_1/3_Sr_2/3_FeO_3_ was measured. (*a*) REXS results at 100 K, which is well below the transition temperature. (*b*) Comparison of the integrated intensity of the elastic scattering at 100 K and 210 K, which are below and above the transition temperatures, respectively.

Fig. 10 ▸ presents the results of resonant X-ray diffraction (RXD) measurements at the Fe *L*-edge of La_1/3_Sr_2/3_FeO_3_(111) thin films obtained using our spectrometer rotation system. This sample was selected to demonstrate the θ–2θ motion commonly used in diffraction experiments and to perform resonant elastic X-ray scattering (REXS) measurements. La_1/3_Sr_2/3_FeO_3_ is known to exhibit magnetic order at low temperatures (Okamoto *et al.*, 2010 ▸), and the RXD/REXS measurement was conducted around ***q*** = (1/6, 1/6, 1/6), where both charge and magnetic orders are present. The scattering vector was set along the [111] direction, and π polarization was used, where the electric field vector of the incident X-ray is parallel to the scattering plane. The photon energy was tuned to ∼710 eV, which corresponds to the peak of the Fe *L*_3_-edge X-ray absorption spectroscopy. The energy resolution was ∼220 meV, and the angular resolution was ∼0.06°, corresponding to ∼0.035 Å^−1^. Since the magnetic ordering begins to form at 195 K, REXS measurements were taken at 100 K and 210 K. Fig. 10 ▸(*a*) shows the REXS results obtained at low temperature, indicating the presence of magnetic order by the increased intensity of the elastic scattering around 2θ = 83°, which corresponds to the periodicity of the magnetic order. Fig. 10 ▸(*b*) shows the integrated elastic scattering intensity below and above the transition temperatures, which is in good agreement with past results (Okamoto *et al.*, 2010 ▸). The fact that the spectrometer adjustment was not performed for each angle scan measurement demonstrates that our rotation system is also highly suitable for REXS experiments.

## 5. Summary

We have successfully developed an XES facility for conducting angle-resolved XES experiments in a facile and effortless manner by implementing the rotation system to the spectrometer of HORNET endstation at SPring-8 BL07LSU. The spectrometer rotation system can rotate the spectrometer to any desired scattering angle without breaking the vacuum and requires no optical adjustment of the spectrometer after the rotation. The rotation mechanism of the sample chamber employs a combination of three rotary stages on which collecting mirrors are mounted. This mechanism not only allows for continuous vacuum connection during the spectrometer rotation but also functions as a mirror adjustment mechanism. It is employed to correct the roll angle deviation of the collecting mirrors within the mechanical twist tolerance of the connecting bellows. Moreover, it can also be used to intentionally move the collecting mirrors out of the optical path, enabling experiments using only the direct path. This sample chamber offers the freedom to move the flange in a wide range of angles, making it suitable for various purposes, including attaching other detectors or cameras for 3D imaging in a vacuum environment, opening up a range of possibilities for future experimentation.

It has been more than ten years since the construction of HORNET, and upgrades are being planned. In line with current best practices, if the vertical source size is focused to 1 µm, the slope error of the grating is reduced to 0.2 µrad, and the spatial resolution of the detector is improved to 5 µm, the spectrometer would be designed to achieve a resolving power of ∼25000 at 700 eV. Since HORNET is relatively small with a total length of ∼2.5 m, the limiting factors are the vertical focus size and the spatial resolution of the detector. Under these conditions, a 10% degradation in energy resolution would be induced by a shift of 0.003° in the roll angle of the collecting mirrors and a change in the height of the spectrometer by 6 µm, or a change in the pitch angle by 0.00068°, which places higher demands on precision. In the future, as the vertical focus size and the detector spatial resolution are improved, smaller spectrometers like HORNET will be capable of achieving even better energy resolution, demanding even higher precision. In such a scenario, the stability of the collecting mirrors mounted on the chamber could become an issue. However, based on the current best practice, both the collecting mirrors and the diffraction grating can achieve sufficient alignment accuracy and stability, and the present rotation system would remain viable after the upgrade. Although our XES facility has many remarkable features, the most exciting feature of our innovation is the dynamic measurements it allows for. Our innovative rotation mechanism enables the spectrometer to slide on metal plates on the granite base during rotation, without floating, offering the potential for continuous measurements when the upgraded spectrometer is combined with a brilliant light source. We are proud to have developed this XES facility, which promises to revolutionize the field of XES experiments and unlock a whole new world of possibilities for researchers.

## Supplementary Material

Overview of the control system. DOI: 10.1107/S1600577523010391/iy5001sup1.pdf

## Figures and Tables

**Table 1:** Optical parameters for the spectrometer at *h*ν = 640 eV

Grating parameters		Geometrical parameters	
Ruled area	60 mm × 20 mm	Vertical size at sample	5 µm FWHM
Curvature	16268 mm	Distance from sample to collecting mirrors	100 mm
Slope error	1.0 µrad in r.m.s.	Angle of incidence to collecting mirrors	88.68°
Diffraction order	1	Angle of incidence to grating	87.1087°
Groove density, *a*_0_	2200 lines mm^−1^	Diffraction angle from grating	−83.8159°
VLS parameters†		Distance from sample to grating	503 mm
*a*_1_	0.603147 lines mm^−2^	Distance from grating to CCD	1963.57 mm
*a*_2_	−3.11735 × 10^−3^ lines mm^−3^	Size of CCD	25 mm × 25 mm
*a*_3_	8.99359 × 10^−6^ lines mm^−4^	Spatial resolution of CCD	24 µm in FWHM
		Angle of incidence to CCD	18°
Collecting mirror parameter			
Size	100 mm × 10 mm		
Curvature	6.0 m		

† The position dependence of the VLS parameter is expressed as *a*(*x*) = *a*_0_ + *a*_1_ *x* + *a*_2_ *x* ^2^ + *a*_3_ *x* ^3^, where *x* is the position on the grating surface and the direction from the incident X-rays to the diffracted X-rays is set to be positive.
