# Supplementary material for: Angle-resolved X-ray emission spectroscopy facility realized by an innovative spectrometer rotation mechanism at SPring-8 BL07LSU
Source: J Synchrotron Radiat. 2024 Feb 1;31(Pt 2):208–16. doi: 10.1107/S1600577523010391 (PMC10914175; doi:10.1107/S1600577523010391)
Supplement: Supplementary file 1 [file s-31-00208-sup1.pdf]

JOURNAL OF SYNCHROTRON RADIATION

Volume 31 (2024)

Supporting information for article:

Angle-resolved X-ray Emission Spectroscopy Facility Realized by Innovative Spectrometer Rotation Mechanism at SPring-8 BL07LSU

Jun Miyawaki, Yuka Kosegawa and Yoshihisa Harada

Supporting Information for
"Angle-resolved X-ray Emission Spectroscopy Facility Realized by Innovative Spectrometer Rotation Mechanism at SPring-8 BL07LSU"

Jun Miyawaki[1,†], Yuka Kosegawa[1], and Yoshihisa Harada[1,2]

[1]Institute for Solid State Physics (ISSP), The University of Tokyo, Kashiwa, Chiba 277-8581, Japan
[2]Synchrotron Radiation Research Organization, The University of Tokyo,
Hongo, Bunkyo-ku, Tokyo, 113-8656, Japan
[†]Present affiliation: Institute for Advanced Synchrotron Light Source,
National Institutes for Quantum Science and Technology, Sendai, Miyagi, 980-8579, Japan

Control System

In order to rotate the spectrometer, it is crucial to synchronize the four motors that control the three rotation axes in the sample chamber and the 2θ rotation of the spectrometer. The difference between the 2θ angle of the connecting flange, determined by the three rotation axes in the sample chamber, and the 2θ angle of the spectrometer can be absorbed by bellows, so it is unnecessary to move them with strict synchronization, and an accuracy of less than 1° is sufficient. However, highly precise synchronization is required for the three rotations in the sample chamber as failure to synchronize can cause the bellows connecting the sample chamber and the spectrometer to twist and break, resulting in a vacuum accident. To prevent such accidents, we developed a control system (depicted in Fig. S1) that integrates control of the three rotation axes in the sample chamber, the spectrometer's 2θ rotation, and an interlock mechanism to detect twist at the bellows. During spectrometer rotation, we moderately synchronize the 2θ motion of the spectrometer with the 2θ motion of the connecting flange in the sample chamber, with a tolerance of 2° for the difference in these 2θ angles. On the other hand, we completely synchronize the three axes in the sample chamber. It is necessary to control the pulses of the three pulse motors as shown in Fig. 7. To achieve this, we divide the curves into small intervals and approximate them with straight lines. Then, by configuring electronic gear settings for the three pulse motors at each interval, we pseudo-operate the curved motion. Inclinometers are installed at both ends of the bellows for the interlock to detect twist and prevent vacuum accidents. Throughout the 2θ rotation, the twist in the connecting bellows remains below the detection limit (less than 0.1°). The control system includes a four-axis XYZθ manipulator for the sample, allowing for operations such as θ-2θ scans. Additionally, the system can change the energy and polarization of the incident X-rays from the beamline, enabling measurements such as incident energy dependence of XES.

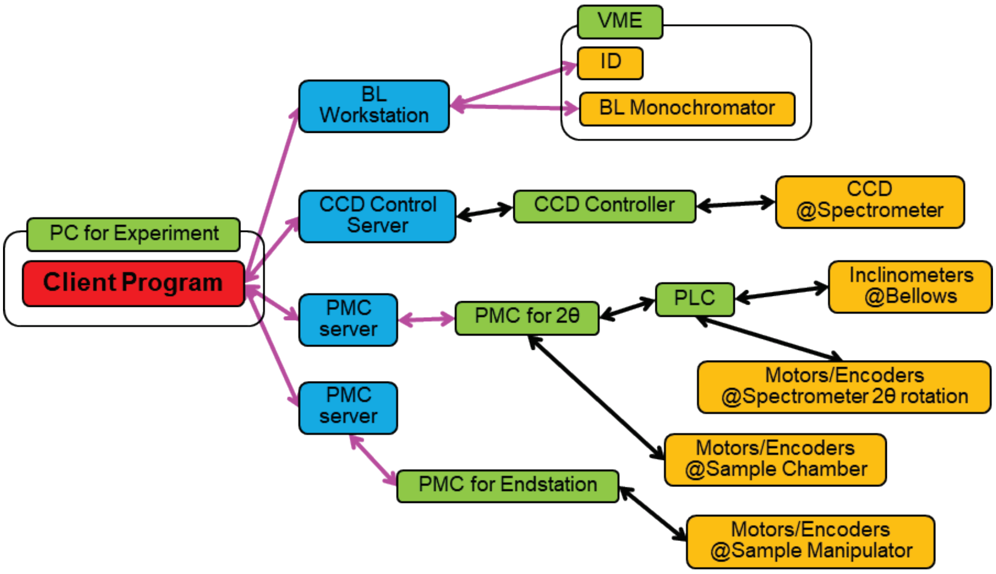

Fig. S1 Overview of the control system. The magenta arrows represent the connection through the LAN, while the black arrows represent the connection through the metal wires.
